# Nurses’ Experiences of Promoting Healthy Aging in the Municipality: A Qualitative Study

**DOI:** 10.3390/healthcare8020131

**Published:** 2020-05-09

**Authors:** Fan Wu, Eva Drevenhorn, Gunilla Carlsson

**Affiliations:** 1 School of Nursing, Peking Union Medical College, Beijing 100144, China; wufan11416@163.com; 2 Department of Health Sciences, Lund University, 221 00 Lund, Sweden; eva.drevenhorn@med.lu.se

**Keywords:** elderly frail, health promotion, primary care nursing, organization

## Abstract

The purpose of this study was to describe nurses’ experiences of promoting healthy aging in municipalities. A descriptive qualitative research design based on semi-structured interviews with 13 nurses was employed. The nurses described the importance of giving older adults the possibility to live as individuals, but also that the organization matters as too does the nurses’ own desire to work professionally and with passion. Nurses in the municipality noticed that in today’s world, there is a changing perspective of older adults. They more often want to continue their previous life and care greatly about quality of life and because of this, they also expect more service from their health care. Our study suggests that nurses should be supported to specialize in elderly care and measures should be taken to reduce the gap between vision and reality when it comes to team work.

## 1. Introduction

It is well-known that many countries experience rapid aging in the population. In Europe, people aged 65 and above account for one fifth of the total population [1]. Because of the decline of physiological and psychological functions in the aging process [2], older adults are more vulnerable to various chronic and acute health conditions and many of them also aging with multimorbidity [3]. Irrespective of disease, healthy aging should be promoted, this being the process of developing and maintaining the functional ability that enables wellbeing in the aging process [4]. That is, health promotion, which is the process of enabling people to increase control over and to improve their health [5] is as important as disease prevention, which focuses on specific efforts aimed at reducing the development and severity of chronic diseases and other morbidity [6].

Nurses in the municipality are the foundation of a country’s primary health care, where they act as health gatekeepers and work in diverse community settings to provide health promotion and disease prevention across the lifespan [7]. In parallel with an increasing aging population and a change in disease spectrum, the role of a nurse is shifting from that of traditionally following physicians’ orders to a more expanded role with responsibility for preventive care [8]. Nurses also pay more attention to health promotion advice, lifestyle counseling, educational programs and the provision of early interventions to prevent exacerbation or complications for persons living with diseases [8,9,10,11]. In a systematic review, unclear professional boundaries, insufficient knowledge and capabilities, and unsupportive organizational and workplace environments were all indicated as barriers to the uptake of new nursing roles in practice [11]. At the health care system level, restricted scope of practice, low compensation levels, and reimbursement policies seemed to limit nurses’ potential to work in expanded roles [12,13].

In Sweden, nurses in the municipality have the key role in taking care of older adults. Because of the limited number of nurses, and a large amount of health care needs requiring qualified practitioners, nurses are facing challenges in fulfilling their role within health care [14]. It should be the top priority to understand the reality and remove the barriers to practice which the nurses are already experiencing in changing roles [15,16]. In this context, the nurses’ own clinical experiences of promoting healthy aging is of importance. Therefore, the purpose of this study was to describe nurses’ experience of promoting healthy aging in Swedish municipalities. 

## 2. Materials and Methods 

A qualitative descriptive approach was applied, using single in-depth, face-to-face, semi-structured interviews [17]. 

### 2.1. Study Setting and Participants

The study took place in three municipalities in the south of Sweden, where nurses in home health care and residential care provided nursing for older adults. A purposive sampling method was applied to recruit the participants [18]. The inclusion criteria were nurses who had at least six months’ experience of working with older adults in the municipality and were able to communicate in English. An effort was made to include a variety of workplaces and genders, and a total of thirteen nurses participated. 

### 2.2. Data Collection

Two of the researchers introduced the study to nurses responsible for the medical issues of home health care in two municipalities and some nurses in a third municipality through personal knowledge. The responsible nurses forwarded the contact information of nurses who showed an interest in participating in the study to the researchers. All the interviewees gave informed consent to participate in the study with reassurance of confidentiality, and the participating nurses decided the time and place they preferred for the interview. 

Data were collected by the first author through face-to-face semi-structured interviews in English from November 2018 to January 2019. All the interviews took place at the nurses’ workplaces in a private room during working hours. Participants signed the informed consent prior to the interview. A self-reported demographic questionnaire (gender, age, job title, education, years of working) was completed by each participant before the interview. Each interview lasted approximately 30 to 45 min. The interviews were audio-recorded with permission from the participants and field notes were taken during the interviews. The interview guide with open-ended questions was developed by the researchers based on a literature review and professional experience. The overarching questions in the interview were about the nurses’ perceptions of the concept of healthy aging, how the nurses associated nursing with healthy aging and the nurses’ experiences of promoting healthy aging. To improve the trustworthiness of the study, two pilot interviews were conducted with nurses, who had experience of taking care of older adults, to test the interview guide and for the interviewer to become used to the interview situation. After each pilot interview, the first and last author reviewed the transcribed interviews in order to ensure that no important points or data were missed related to the study aim. In addition, a Swedish–English dictionary was developed with some common professional titles and words used in primary care, as some words in English were not always familiar to the Swedish-speaking nurses. The nurses were also able to express some words in Swedish to clarify what they meant during the interviews, but this option was used only on a few occasions.

### 2.3. Data Analysis

The interviews were transcribed verbatim by the first author. All transcripts were checked for accuracy by the last author and any identifiable information was removed prior to analysis. Content analysis was used to analyze the data, as described by Graneheim and Lundman [19]. Three steps were included in the process. First of all, reviewing the whole text of the interviews to obtain an outline of the content. Secondly, dividing the text into meaning units. Then, condensing the meaning units and labeling them with codes. These codes were compared and grouped into sub-categories and then into categories (Table 1). To assert the credibility of the analysis, team meetings were held regularly to discuss the coding process and refine the sub-categories and categories.

### 2.4. Ethical Considerations

This study followed the principles outlined in the Declaration of Helsinki [20]. All participants were informed of the purpose of the study and the amount of time required for participation. Informed consent was signed. Participants were assured that their personal information and interview responses would remain confidential, and they could withdraw from the study at any time without giving any explanation.

## 3. Results

In total, thirteen nurses participated and they had between one and twenty-five years of working experience (Md = 11) in home health care or in residential homes in the municipality. Ten of them were women and three were men with a median age of 50 (min-max: 33–63) years. Six of the nurses worked in home health care, and seven worked at residential homes. Eleven of them worked with older adults on a daily basis and two were managers of residential homes. All of the participants were registered nurses. Of them, five had special education for working in primary care and another two had special education for other areas of care. Finally, two of the participants were heads of nurses in the municipality.

During the analysis of the nurses’ experiences of promoting healthy aging three categories and nine sub-categories emerged (presented in Italics in the text). The categories were: (1) Older adults should be given the possibility to live as individuals; (2) Organization matters; (3) To work professionally and with passion (Table 1).

### 3.1. Older Adults Should Be Given the Possibility to Live as Individuals

Nurses described that the nature of promoting healthy aging was to support older adults to live independently and to maintain their ability to live as well as possible. They described that it was important for them to help the older adults to live in a way that the older adults themselves preferred.

*They want some level of independence, to be able to decide what they want to do. Even if they can’t do everything they want. They want to maintain that independency.* (#6)

In order to shoulder the responsibility as a nurse to promote healthy aging, they felt that it was important to listen to the older adults in order to know about the needs and expectations of their health.

*I see the whole person. When I meet older people, and have to help them with something-you know, if they get any sort of injury-and I do the bandage and wound care, then I don’t just look at that, I ask the person: How are you today? You know, this conversation is very important.* (#12)

Health was expressed as not just the opposite of disease, but as also concerning life and social wellbeing. The nurses spoke about the necessity of seeing the whole picture of older adults’ health, life and habits, even if their habits were sometimes less healthy.

*Life quality for her is to enjoy candy.* (#4)

Having a good understanding of older adults made it easier for the nurses to promote healthy aging. The nurses tried to consider the older adults’ ideas and willingness in order to provide nursing care that preserved the remaining abilities and prolonged independent living, so as to improve the older adults’ quality of life.

*I think it is important to not make people sicker than they are. To preserve everything they can do themselves. Not take over and do a lot of things for them.* (#5)

Nurses also noticed that the perspectives on health and aging are changing among older adults over time. Today, older adults more often want to continue their previous life and care greatly about their quality of life and the meaning of life. Because of this, they expect more from health care services.

*Because they know how to live, they have lived long lives, explorative lives. They don’t want to stop because they are sick. So I think we have a really tough challenge, and we are going to be more challenged every year.* (#1)

However, nurses also felt challenged by those older adults who did not participate in health promoting programs held by nurses in home health care or at the residential homes. Some of the older adults tended to seek help when they were sick, but they were not aware of their responsibility for their own health at an earlier stage. Some expected society to take care of them; it was the type of mentality they had. That the older adults’ relatives have a role in promoting health aging was also mentioned as important. Nurses reported that some of the relatives were supportive and could accompany older adults, and help the nurses when they made assessments for nursing plans and interventions.

*They have a big role. Because, for example, people who live here, their relatives or children can tell me much about them. For them it’s natural, but it helps me get closer to the patient. Because they can tell a lot about their earlier lives, for example, what they have been doing and what they like. Communication with their children is really important.* (#9)

However, nurses also expressed that some relatives either ignored their role in helping the older adults to achieve psychological wellbeing or interfered too much with the nursing. These relatives were considered to be challenging to deal with.

*Relatives who don’t visit the old people so much, they are the most demanding. Some relatives visit every day, and they can be very demanding. But some come once a week and they are just laid back and don’t say that much. But then we have the people who have not seen their mother in three years, and then she is suddenly going to die, then they get upset: no, you have to save her, you have to give her everything. They can be a challenge. They are not in the same phase [as they would have been if they had visited their mother more often].* (#6)

The nurses expressed that there should be limits to the relatives’ interference. In this respect, they mentioned the importance of keeping a balance in the relatives’ participation in the care, and to always keep in mind that the older adults’ will and ideas come first.

### 3.2. Organization Matters

The nurses expressed that promoting healthy aging was complex and challenging work, and they could not do everything for the older adults, like for example, changing the amount of medication. Health professionals had to cooperate with each other in some situations to help older adults achieve well-being. Teamwork was important but not easy. Some nurses expressed that within the team they were acting rather like the “spider at the center of the web”. However, nurses indicated that it was difficult to form a team and there might be several reasons for this. One was that the number of available health care staff was limited. Another reason was that the staff in the health care system and in the social care system had different perspectives and goals and had different laws and regulations to follow.

*My organization has one administrative manager. And the other [the nurse aide] has another. And we have the same patients, the same older people. So, it’s difficult to work as a team, when I can do something but the others can’t. To work as a team, you have to do the same thing.* (#7)

The nurses also noticed that changing to a more promotive way of working required an open mind. Promoting healthy aging was a forward-looking and ongoing work, but the nurses felt discouraged when the manager only focused on illness and had a negative response to preventive and promotive work among older adults. Nurses thought that staff in the higher positions should have a greater vision and be open for and become more actively engaged in changes.

*But I still think that the health care itself is too task oriented. They [the managers] don’t see the benefit of preventive, or holistic care. We don’t have the resources. They are always talking about the resources, but I think this is short-term thinking. If you see that there are things you can prevent, then you can save a lot of suffering and time and money for the society. But sometimes it’s just like talking to a brick wall.* (#8)

*And I have had different managers in the organization throughout my work life and not everybody has been good at that [seeing the benefits of preventive work]. Because some people just want to come and do their job. And that’s ok. We need those people who come and do their job. But we also need people who are thinking ahead, have ideas, want to try new things. For that to happen-because we need it to happen, because we cannot work in the same way-we need minds that are open to change.* (#4)

Most of the time, nurses had to reach the older adults through other members of staff. They could not be as directly involved in the caring as they wanted to due to their heavy workload. Recurrent barriers mentioned, that hindered the nurses’ caring work, were burdensome administrative tasks, limited numbers of nurses and high staff turnover. So, most of the time the nurses relied on the nurse aides and considered the nurse aids as their eyes and ears in making the right nursing decisions.

*Since I always stand a little bit on the outside. It’s not really… I have to rely on the nurse aides. They have to tell me what they feel about the patient, and I have to listen to them, to help me make the right decisions.* (#3)

*And it’s very frustrating from the nurse’s side, to know that your education-research tells you what you should do, the way you should do things, but the human resources are not actually in place, are not always enough to do that.* (#11)

### 3.3. To Work Professionally and with Passion

Nurses expressed that they were motivated to work with older adults in the municipality by the sense of the value of their work. Some nurses had chosen to work with older adults in home health care because of the free and flexible working conditions. It was encouraging for them when they felt that their work made a difference to the older adults, their families and the whole society.

*When I see a difference in how a person feels, it gives me strength and inspires me to carry on with my job.* (#9)

*And for society too. It’s a good upward spiral. It influences the whole society, so society itself develops positively. And if we do a good job, then it would help the society. So maybe you can save money, for example, save money for the municipality or district.* (#2)

The nurses emphasized the necessity of being up to date with their medical knowledge, so they could carry on being a good nurse when facing the complex situations of the new era. This was because the number of aged people was on the rise, and the disease spectrum was changing over time.

*And, of course, some sort of training is necessary, I think. So it’s good to have that as well, at least one week a year when you can study more, learn about new things. And that can be very creative and stimulating too. If you find out something new and want to try it out. I think that’s an important part of being a good nurse.* (#3)

The nurses made a number of efforts to keep themselves up to date in different areas and developed their knowledge of evidence-based nursing. They also mentioned that communicating with other nurses and health care providers could help them to broaden their horizons as regards taking care of older adults.

*I read a lot of articles, for example. I keep myself very up to date, and I think that is very important. So you know all the new research and so on. Because if you know the new research, you become curious, you won’t accept the routine. So you can always make it better than it is.* (#1)

*You meet other people; you meet other nurses. You can hear about their patients, and every time you hear someone talking about the patients, you learn something.* (#1)

The nurses also emphasized their professional role in promoting healthy aging, how they were exercising their profession. It was not only about skills in managing different diseases, but also their individual personalities. They had to be sensitive, thoughtful and nice but also assertive enough to insist on their professional view when they found that the older adults’ or their relatives’ ideas could be more harmful than beneficial.

*So, I have some words here I think. Empathy, sensibility, thoughtfulness, compassion, you have to have a degree of humility when dealing with people.* (#12)

*And you have to know when to be tough, and when to be soft. Because some patients need a firm nurse. So you need to have that personality that you cannot be just nice all the time, you also have to be firm.* (#1)

However, there were nurses who were worried that the nature of nursing was disappearing. They thought that nurses nowadays were doing medical work rather than caring. They had lost their awareness of identifying problems and solving problems from a nursing-perspective.

*Medicine is what the doctor tells the nurses to do, it is not what you find is needed to be done. And the nurses don’t do that [what they find is needed] anymore. They just do what doctors tell them to do. They have no time to do anything else, I really think so.* (#11)

## 4. Discussion

Our findings of nurses’ experiences of promoting healthy aging in the municipality revealed the importance of giving older adults the possibility to live as individuals in order to maintain a good life in the aging process, but also that the organization matters as too does the nurses’ own desire to work professionally and with passion. 

The nurses in our study defined their role in promoting healthy aging as being a support to older adults to live the way they preferred as an independent person. This is in line with the model of person-centered care, which means not only treating older adults so that they are able to maintain their physical, psychological, cognitive and social wellbeing, but also focusing on improving autonomy in elderly care [21]. Comparable to our findings, the nurses in a study by Kim and colleagues [22] emphasized the importance of performing professional nursing assessments to see the individuals’ abilities so as to prolong the older adults’ independent living. However, nurses in our study felt that one of the challenges was that older adults’ perspectives had changed over time. Nowadays, older adults have a variety of attitudes toward aging and health. There might be a distinction between relatively active older adults and older adults who are very dependent on care and support [23]. Older adults that are very dependent may also want to continue their previous life even if they are sick. The nurses reflected on this tough challenge that might increase every year and their need to update their own body of knowledge to deal with these different needs. 

With the development of society, the relationship between social ties and health outcomes becomes increasingly prominent especially in the aging population [24]. Some relatives were regarded as being a positive support for the older adults to achieve health and to buffer stressors in later life [25]. A previous study showed that family support was reported to be the most influential factor to life satisfaction among older adults [26]. However, in this study, the nurses expressed that some of the older adults’ relatives hindered the nurses’ work, which might lead to negative health outcomes for the older adults. This is consistent with what Litwin and Stoeckel [27] discovered about family networks, that they were not always facilitative for mobility improvement where some relatives asked too much just from their own perspective and ignored the needs of the older adult. There were also relatives who over-compensated in the care they gave, which resulted in the older adult being discouraged from taking the necessary steps to maintain abilities and overcome limitations. These findings indicate that nurses in the future, through their professional perspective should assess the older adults’ family relationships to balance older adults’ and their relatives’ will. Another challenge that the nurses experienced was the gap between vision and reality, when it came to teamwork building. The nurses wanted the managers to be more open minded to the importance of disease prevention and health promotion for older adults. Multidisciplinary teamwork is important in primary health care due to the complicated health needs of older adults [28]. In Sweden, however, this is a problem as the number of health care professionals is limited. In this study, the nurses in home health care mentioned their feelings of loneliness and stress at their workplace as they could not manage all the complex problems around the older adults and had to consult the general practitioner (GP). However, the older adults sometimes had to wait for a long time to get proper help as the GP is employed by the county council, whereas the nurses in home health care are employed by the municipality. This made it difficult for the nurses to get in touch with the GP, for example, when the issue was left unattended on the GP’s secretary’s desk. Moreover, the staff in the team caring for older adults were members of different organizations within home health care and thereby had to follow different rules and regulations, especially nurses and nurse aides. Nurses described that when they cared for the same older adult there could be situations where the regulations of the health care system and social care system were contradictory. Teamwork is important in buffering the work-related stress and reducing negative feelings of loneliness and alienation [29]. Therefore, there is a need for the managers of the organization to create an environment where nurses have easy access to GPs, while ensuring effective health service delivery. Managers also should be aware of how to coordinate the different regulation systems. 

The core vision of Swedish primary health care is to provide holistic and comprehensive care, including health promotion, disease prevention and curative care to rehabilitation [30]. However, in our study the nurses’ work was restricted due to the gap between this good vision and practical work as the managers in the organization did not always prioritize the nurses’ work in health promotion and disease prevention among older adults. Most of the nurses’ work was considered to be task-oriented and a great amount of resources were devoted to the treatment of geriatric diseases. The nurses saw themselves as the spider in a web as they needed to contact people in different departments to get the help and service to function satisfactorily in the daily care of the older adults [31]. Nurses in this study expressed that the burdensome administrative work hindered them in working closer to the older adults. This is also in accordance with earlier findings, which show that registered nurses in municipal elderly care experienced a high level of time pressure [10]. The organizational structure matters for promoting healthy aging in primary health care. Managers in the Swedish organization should then consider optimizing and simplifying nurses’ office work and contacts within the team so that they work in a more efficient way. Moreover, it seems essential for different stakeholders to discuss how to shift the focus from disease treatment to disease prevention and health promotion for older adults, and how to collaborate with each other to improve the care in the reality of daily practice. This interdisciplinary collaboration has to start already during education [32].

In this study, the nurses insisted on the preventive work because of their passion and personal interest in caring for older adults, especially awareness of their valuable work, which was a result of the positive feedback they had from older adults and their relatives. This is exemplified by the findings from Aparicio and colleagues [33], who found that gratitude from older adults and their families could contribute to motivation for taking care of the older adults and retention among nurses. In our study, keeping up to date over time was important for the nurses, in order to be able to face complex situations. They wanted their work to be of high quality and they tried to implement innovative practices [31]. When it came to the nurses’ daily practice, staff shortages and staff turnover were two main problems. By giving the nurses the opportunity to take specialized courses to keep updated, their feeling of being able to give care of high quality, and being a valuable member of the team might support them to keep working in the municipality. It may also contribute to reduce the high staff turnover if the managers could arrange a specific internship plan for the newly employed nurses, to support them in the complex situations they face in the municipality. Another key point, which was mentioned by the nurses, was to not forget the nature of caring. Nurses should not only deal with the ordered medical treatment, but also do the nursing assessment and make care plans to solve the present or potential problems among the older adults.

### 4.1. Methodological Considerations

The research group consisted of researchers with clinical experiences in the area of nursing, healthy aging, occupational therapy and qualitative research methodology. Further, to ensure trustworthiness [34], a study specific interview guide was used, information-rich participants selected and a method for data collection used, allowing the information given by nurses to be accurately interpreted. All nurses were happy to talk about the subject and shared in detail their experiences. The first author, fluent in English, conducted all the interviews and the second and third author with Swedish medical and cultural background assisted in data collection and analysis. Before the nurses included in this study were interviewed, the first author conducted two pilot interviews with nurses with special education for working in primary care and got feedback from the nurses and the co-authors. The only problem identified was that even if the nurses were fluent in English, they were not familiar with the English wording of all Swedish professionals’ titles and words used in primary care. To deal with this limitation, we developed a Swedish–English dictionary as a guide, but they only used this on a few occasions. They could express single words in Swedish to clarify what they meant during the interviews, but also this occurred only on a few occasions. The limitation, due to language and translation problems, was also partly balanced by the mother tongue for two of the researchers (GC and ED), which is Swedish. Furthermore, credibility of findings was strengthened through all authors being involved in the analysis of data. Several quotations are used in the text so the reader has the ability to judge the credibility of our study. Regarding the transferability, a purposive sampling method was used with variation in age, sex, years of working, workplaces and qualifications and we consider that our sample is acceptably heterogeneous. Another limitation might be that the study was conducted in only three municipalities, which means that there might be a risk that the result has captured just local practices. However, the health care system is almost the same in municipalities all over Sweden.

### 4.2. Clinical Evidence

Healthy aging is about having a good understanding of older adults and the necessity of seeing the whole picture of their health, life and habits. The teamwork is already challenging, but with new generations of patients and new expectations on service from their health care, this will increase. The organization and administrative workload matter for health promoting work. The managers’ coordination of the team members and education that support nurses specializing in elderly and primary care is also of high importance in promoting healthy aging. 

## 5. Conclusions

This study highlighted possibilities and challenges for nurses to promote healthy aging in the municipality. The nurses reported how they seek for giving older adults the possibility to live as individuals in order to maintain a good life in the aging process, but the changing perspectives of older adults and proper involvement of relatives sometimes challenged their work. They also mentioned that they could not be as involved as they wanted due to heavy administrative workload and some reflected upon not forgetting the nature of caring. The organization they work in matters as well as the nurses’ own desire to work professionally and with passion. The study suggests that, when it comes to teamwork, resource deployment, and coordination of the health care system and social care system, measures should be taken to reduce the gap between vision and reality. This requires managers that are open minded to the idea of advancing the promotion of healthy aging and disease prevention in older adults, and support nurses in promoting healthy aging.

## Figures and Tables

**Table 1:** Nurses’ experiences of promoting healthy aging-an overview of categories, sub-categories, and code examples.

Category	Sub-Category	Code Examples
Older adults should be given the possibility to live as individuals	Support older adults to live independently	To maintain independence To treat old people as individuals
	Perspectives on health and aging are changing among older adults	Changed views of health To feel needed
	Relatives have a role in promoting healthy aging	Easier to involve family members Work with family is challenging
Organization matters	Teamwork is important but not easy	Common goals Two systems
	Change to a more promotive way of working requires an open mind	No time for proactive work Change of philosophy
	Reach the older adults through other members of staff	Heavy administrative workload Rely on the nurse aides
To work professionally and with passion	Sense of valuable work	Positive feedback Like to work with old people
	Necessity of being updated	Gain new knowledge Communicate with others
	Exercising one’s profession	Skills are important Open to complex situations
